# Bisphosphonates as Radiopharmaceuticals: Spotlight on the Development and Clinical Use of DOTAZOL in Diagnostics and Palliative Radionuclide Therapy

**DOI:** 10.3390/ijms25010462

**Published:** 2023-12-29

**Authors:** Céleste Souche, Juliette Fouillet, Léa Rubira, Charlotte Donzé, Emmanuel Deshayes, Cyril Fersing

**Affiliations:** 1 Nuclear Medicine Department, Institut Régional du Cancer de Montpellier (ICM), University Montpellier, 34298 Montpellier, France; 2 Institut de Recherche en Cancérologie de Montpellier (IRCM), INSERM U1194, University Montpellier, Institut Régional du Cancer de Montpellier (ICM), 34298 Montpellier, France; 3 IBMM, University Montpellier, CNRS, ENSCM, 34293 Montpellier, France

**Keywords:** DOTAZOL, bisphosphonates, bone metastases, theranostics, nuclear medicine, ^68^Ga, ^177^Lu

## Abstract

Bisphosphonates are therapeutic agents that have been used for almost five decades in the treatment of various bone diseases, such as osteoporosis, Paget disease and prevention of osseous complications in cancer patients. In nuclear medicine, simple bisphosphonates such as ^99m^Tc-radiolabelled oxidronate and medronate remain first-line bone scintigraphic imaging agents for both oncology and non-oncology indications. In line with the growing interest in theranostic molecules, bifunctional bisphosphonates bearing a chelating moiety capable of complexing a variety of radiometals were designed. Among them, DOTA-conjugated zoledronate (DOTAZOL) emerged as an ideal derivative for both PET imaging (when radiolabeled with ^68^Ga) and management of bone metastases from various types of cancer (when radiolabeled with ^177^Lu). In this context, this report provides an overview of the main medicinal chemistry aspects concerning bisphosphonates, discussing their roles in molecular oncology imaging and targeted radionuclide therapy with a particular focus on bifunctional bisphosphonates. Particular attention is also paid to the development of DOTAZOL, with emphasis on the radiochemistry and quality control aspects of its preparation, before outlining the preclinical and clinical data obtained so far with this radiopharmaceutical candidate.

## 1. Introduction

Breast, prostate and lung are the solid cancers with the highest incidence of bone metastases [1], which can lead to complications such as severe bone pain, pathological fractures, nerve compression, hypercalcemia and overall morbidity [2]. Since the 1970s, nuclear medicine has played a key role in the diagnosis of bone metastases with high-sensitivity molecular imaging approaches such as whole-body bone scintigraphy [3,4,5,6]. Furthermore, the discipline is well-suited to the concept of theranostics, which refers to the convergence of diagnostic and therapeutic procedures by using the same molecular platform combined with either a photon-emitting radioelement (diagnostic) or a particle-emitting radioelement (therapeutic) [7]. In this context, the most appropriate molecules for bone matrix targeting tend to be bisphosphonates; as a matter of fact, the simplest ^99m^Tc-labeled derivatives of this chemical family, i.e., medronate and oxidronate, have been extensively used as bone single-photon emission computed tomography (SPECT) imaging agents for over 40 years [8]. Therapeutic counterparts to these SPECT tracers have been designed and studied in humans, such as [^188^Re]Re-etidronate, which showed some potential in terms of efficacy [9,10,11,12,13,14,15,16,17,18,19,20,21] and improvement of patients’ quality of life [22]. However, rhenium’s complex redox chemistry hampered the expansion of this therapeutic option, as it is challenging to implement in a conventional radiopharmaceutical environment [23]. Likewise, rhenium-188 can be obtained from a ^188^W/^188^Re radioelement generator, but no fully pharmaceutical-grade generator is currently available [24], causing preference to be given to “simpler-to-use” radioelements.

**Figure 1:**
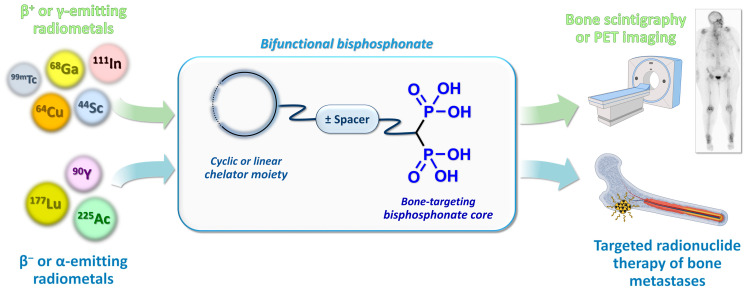
General principle of a theranostic approach in nuclear medicine, applied to bifunctional bisphosphonate derivatives.

In connection with the ever-growing development of a wide variety of radiotracer candidates for PET imaging, the use of ^68^Ga has recently become increasingly popular, owing to its availability via GMP-grade ^68^Ge/^68^Ga generators [25] and to its rich yet sufficiently straightforward radiochemistry [26]. With regard to therapy, ^177^Lu has lately emerged as a key radioelement, with a 6.7 day half-life (allowing the easier logistics of production and supply of either the radioisotope alone or radiolabeled molecules); low-energy beta particles (E_β_(max) = 497 keV [78.6%], 384 keV [9.1%] and 176 keV [12.2%], theoretically allowing the irradiation of small lesions and decreased damages to non-target tissues because of the short beta particles range); and radiochemical properties that are now largely documented and mastered [27]. To date, the ^68^Ga/^177^Lu theranostic pair has demonstrated its clinical reliability with FDA-approved radiopharmaceuticals in the management of neuroendocrine tumors (DOTATOC and DOTATATE vector molecules) [28,29] and metastatic prostate cancer (PSMA ligands vector molecules) [30,31]. This ^68^Ga/^177^Lu approach was then transposed to bone targeting with the use of chelator-bearing bisphosphonates, either originally conceived as MRI contrast agents or directly designed as nuclear medicine theranostic agents. Depending on the nature of the chelating agent functionalizing the bisphosphonate, several radioelements could be complexed by these bifunctional molecules, either for diagnostic or therapeutic purposes (Figure 1) [32]. Among these chelator-bearing bisphosphonate, DOTAZOL is one of the most recent derivatives to have been used on humans, for both PET imaging with [^68^Ga]Ga-DOTAZOL and targeted radionuclide therapy (TRT) with [^177^Lu]Lu-DOTAZOL.

After an initial overview of the main chemistry and biology aspects of bisphosphonates and their use as radiopharmaceutical agents, this review will provide a summary of the preclinical development of several chelate-conjugated bisphosphonates to finally focus on DOTAZOL, with a particular emphasis on the chemical and radiochemical aspects. DOTAZOL’s evaluation and use on humans, for both diagnostic and therapeutic indications, will be discussed with a view to defining the current and possible future roles of this radiopharmaceutical in PET diagnostics and the TRT of bone metastases.

## 2. Bisphosphonates: From Conventional Drugs to Radiopharmaceuticals

### 2.1. Development and Pharmacology Basics of Bisphosphonates

Before their use as medicinal products for treating bone or calcium metabolism disorders, bisphosphonates were originally designed as softeners and complexing agents for textile, food and oil industries [33] dating from their first synthesis in 1865 [34]. Initial biological applications of bisphosphonates were related to their structural analogy with inorganic pyrophosphate, which confer in vitro and in vivo inhibitory properties on bone resorption, combined with resistance to acid- or phosphatase-catalyzed hydrolysis [35,36]. These effects on bone were mainly mediated by the inhibition of the hydroxyapatite crystal dissolution but were associated, for the earliest bisphosphonates derivatives, to the inhibitory properties of skeletal mineralization [37]. This was therefore one of the characteristics to be controlled as successive bisphosphonate analogs were developed.

**Figure 2:**
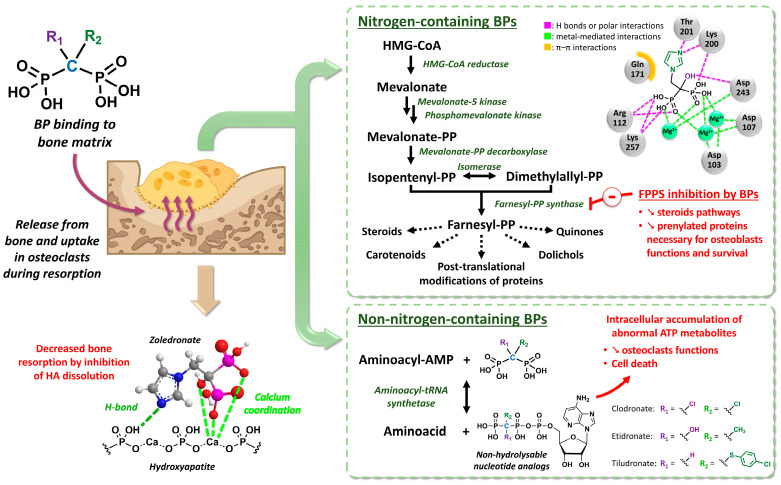
Extracellular and molecular mechanisms of the action of bisphosphonates, exemplified with zoledronate for nitrogen-containing BPs. Molecular interaction representations between zoledronate and farnesyl-PP synthase were adapted from schematic 2-D features of protein-ligand complexes from the Protein Data Bank (PDB) [67]. BP: bisphosphonate; HA: hydroxyapatite; PP: pyrophosphate.

The chemical structure of bisphosphonates is centered on the P-C-P pharmacophore, which is responsible for inhibiting bone resorption, whereas several analogs such as ethilenediphosphonates (P-C-C-P) or iminodiphosphonates (P-N-P) are inactive. This chemical motif is responsible for high affinity and strong binding to hydroxyapatite crystals in the bone mineral matrix; in addition, the central sp^3^ carbon offers numerous pharmacomodulation possibilities by introducing a variety of substituents in this position. The natures of the residues on this central carbon (referred as R_1_ and R_2_) allow the identification of several groups of molecules, the first being the non-hydroxy- and non-nitrogen-containing bisphosphonates groups, originally headed by clodronate [38]. Since the late 1960s, it has been demonstrated that derivatives containing a R_1_ hydroxy group on the central carbon exhibited improved hydroxyapatite binding, with better blockage of hydroxyapatite crystal dissolution and growth, combined with a greater affinity for calcium (particularly in the bone mineral matrix) [39]. The first hydroxylated bisphosphonate to be used on humans was etidronate, which was originally synthesized in the late 19th century [40] and first used as a calcification inhibitor in the mid-1970s to treat patients with fibrodysplasia ossificans progressiva [41]. In addition to their binding to the mineral components of the bone, an intracellular action of bisphosphonates related to etidronate and clodronate has been identified, based on their structural homology with inorganic pyrophosphate. Such molecules could be incorporated in nucleotide precursors to form non-hydrolysable analogs [42,43,44,45], which could then accumulate in osteoclasts and lead to cell death (Figure 2) [46,47,48]. It was then evidenced that, with R_2_ being a basic primary alkylamine as in pamidronate and alendronate [49,50], antiresorptive effects were 10 to 100 times higher than with the non-nitrogen-containing derivatives. This suggested alternative specific intracellular effects associated with the R_2_ nitrogen chemical moiety [51]. Notably, N-containing bisphosphonates displayed inhibitory properties against several enzymes involved in the mevalonate biosynthesis pathway, contributing to sterol production, especially farnesyl-pyrophosphate synthase [52,53]. Thus, because of the critical role of the mevalonate pathway for osteoclastic functions, this mechanistic aspect of nitrogen-containing bisphosphonates is of major relevance for this cell type (Figure 2) [54,55,56,57,58]. The importance of the nitrogen atom in the R_2_ side chain appeared to be even greater as a tertiary amine motif further increases potency (e.g., in ibandronate) as well as the inclusion of the nitrogen in an aromatic ring (e.g., a pyridine in risedronate or an imidazole in zoledronate) [59]. In addition to their effect on osteoclasts, some bisphosphonate derivatives appear to have a beneficial effect on osteoblasts, enhancing their inhibitory properties on osteoclastogenesis [60,61] and inducing proliferative [62,63,64,65] or anti-apoptotic effects [66].

**Figure 3:**
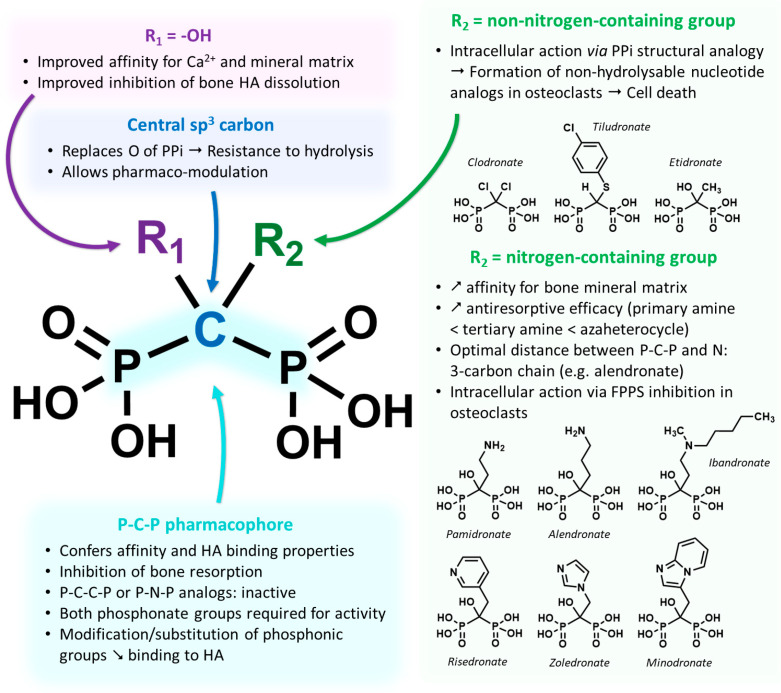
Main structure-activity relationships among bisphosphonates and the relevant chemical structures of bisphosphonates used for clinical applications. FPPS: farnesyl-pyrophosphate synthase; HA: hydroxyapatite; Ppi: inorganic pyrophosphate.

Overall, thorough investigations of bisphosphonates structure-activity relationships, with the significant contribution of crystallographic studies [68], have led to a better understanding of the main mechanistic aspects of these compounds [69] and has opened the way to potential novel utilizations [70,71,72]. Figure 3 summarizes the key medicinal chemistry elements outlined in bisphosphonate series. To date, depending on the country, around 10 bisphosphonates for pharmacological uses have been approved for clinical indications in humans (almost all being hydroxy- and nitrogen-containing compounds). Their main applications are the treatment of post-menopausal osteoporosis, the management of osteolysis and hypercalcemia of malignant origin, the prevention of bone complications (fractures and spinal cord compression) in cancer patients and the treatment of Paget’s disease. The safety profile of bisphosphonates is well-defined and the most serious adverse affects are either rare or predictable, including gastrointestinal (nausea, vomiting, epigastric pain, esophagitis, gastric ulcer and dyspepsia), kidney (renal failure if given in IV), musculoskeletal (pain, atypical femoral fractures and osteonecrosis of the jaw) or cutaneous side effects. Some of the most recent drugs, such as zoledronate, tend to be positioned as first- or second-line therapies, given their efficacy and convenient treatment regimens (injectable drugs are characterized by 12- or 18-month dosing intervals, which ensures compliance). Their use as adjuvant therapy in selected malignancies such as breast cancer [73,74,75,76] shows positive effects, although small, on bone recurrence, fracture rates, cancer mortality and overall survival [77], possibly via their ancillary effects on cancer cells including immunomodulation and synergy with anticancer agents [78]. Moreover, beyond their pharmacological utility, selected bisphosphonates are currently used or investigated as bone molecular targeting agents for nuclear medicine applications.

### 2.2. Bone Metastases Molecular Targeting and Early Bisphosphonate-Based Radiopharmaceuticals

**Figure 4:**
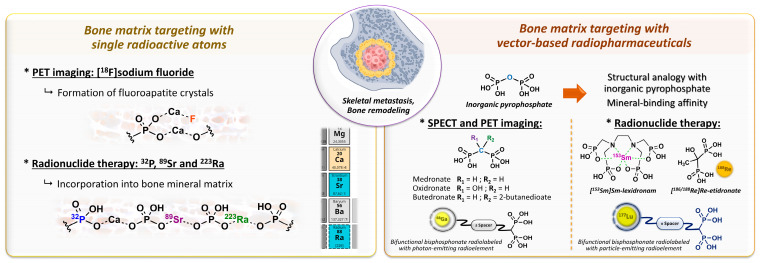
Overview of radiopharmaceuticals for the diagnosis and treatment of bone metastases, depending on their targeting pattern.

**Figure 5:**
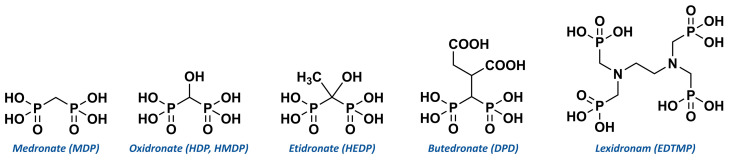
Chemical structure of monofunctional bisphosphonates and related agents for ^99m^Tc (MDP, HMDP, HEDP, DPD), ^188/186^Re (HEDP) or ^153^Sm (EDTMP) radiolabeling.

Bone microenvironments provide a privileged metastatic niche, especially when tumor cells display a particular affinity for bone tissue [79]. In several diseases such as lung cancer, bone metastases are associated with predominant osteoclast-mediated bone resorption lesions which are described as lytic. Conversely, prostate cancer patients with bone metastases often exhibit dense osteosclerotic lesions with increased osteoblast activity [80]. Mixed lesions are prevalent across various tumor types but are especially frequent among patients with metastatic breast cancer [81]. In nuclear medicine, several bone-addressed radiopharmaceuticals have therefore been developed for diagnostic or therapeutic purposes, exploiting two main principles for targeting bone metastases (Figure 4):

- Specific bone targeting based on the electronic properties of a free radioactive atom: two isotopes of the same atom do not differ in their electronic structure and therefore have identical chemical properties, which allows skeletal targeting by the isotopes of atoms with natural affinities for bone. This applies to [^18^F]fluoride ions, which replace hydroxide anions in hydroxyapatite crystals to form fluoroapatite in the bone mineral [82]. The [^18^F]NaF PET imaging agent tends to be more accurate than bone scintigraphy for the detection of skeletal metastatic lesions in several types of cancer [83,84,85,86]. In therapy, [^32^P]orthophosphate (t_1/2_ = 14.3 days; E_β-max_ = 1.71 MeV) is also incorporated into hydroxyapatite crystals [87] and was historically used to treat painful osteoblastic metastases [88]. This treatment showed moderate efficacy with the disappearance of pain symptoms in almost half of patients [89] but caused common bone marrow toxicity, especially in patients with renal impairment. Likewise, the chemical elements in the same group/column of the periodic table of elements are characterized by the same number of valence electrons, and therefore, by usually comparable chemical reactivity. Part of the same group as calcium, ^89^Sr (t_1/2_ = 50.5 days; E_β-max_ = 1.49 MeV) is an alkaline earth metal that accumulates in lesions with high osteoblastic activity and has been used under its dichloride salt form in the palliative treatment of pain associated with bone metastases [90], especially in prostate cancer. The initial clinical trials with this radiopharmaceutical in monotherapy showed modest effects on pain control in bone metastases associated with substantial bone marrow toxicity [91,92,93], while its use in patients treated with doxorubicin [94] or docetaxel [95] tended to improve both the symptoms associated with bone metastases and survival. Similarly, alpha-emitting radionuclide ^223^Ra (t_1/2_ = 11.4 days; Eα = 5.0 to 7.5 MeV [95.3%]; E_β-max_ = 1.37 MeV and 1.42 MeV [3.6%]) showed an overall survival benefit in patients with metastatic prostate cancer, with a significant 9-month delay in bone-related events when associated with a bone-protecting agent (e.g., denosumab) [96]. To date, radium-223 dichloride (Xofigo^®^, Bayer, Leverkusen, Germany) is the only FDA- and EMA-approved targeted alpha therapy available.
- Specific targeting based on a vector molecule with bone tropism: in scintigraphic imaging, medronate (MDP) [97] and oxidronate (HMDP) [98,99] were among the first bisphosphonates to be used as bone scintigraphy imaging vectors on humans after the pioneering application of [^99m^Tc]Tc-etidronate [100,101,102,103] (Figure 5). These two compounds are characterized by their simple chemical structures, which do not contain dedicated chelation sites. Although their formulation in single-vial cold kits for radiopharmaceutical preparation makes ^99m^Tc radiolabeling simple and ensures high radiochemical purity levels, [^99m^Tc]Tc-MDP and [^99m^Tc]Tc-HMDP complexes do not form a single defined chemical entity but are rather structured into a mixture of monomers, oxo-bridged dimers and oligomeric clusters of varying sizes, featuring diverse technetium-oxo core arrangements, oxidation states and ligand coordination numbers [104] with a composition that varies according to pH, technetium concentration and oxygen amount [105]. Lastly, the phosphonate groups of MDP and HMDP (as well as the hydroxyl group of HMDP) serve both as coordination sites with ^99m^Tc and as recognition sites for the bone mineral matrix. Consequently, the bone affinity of the corresponding ^99m^Tc complexes is intrinsically reduced [106]. Even so, these radiopharmaceuticals remain reference bone scintigraphy agents, either in oncology for cancer staging [107,108] and therapeutic response evaluation [109,110,111] or in benign bone disorders such as Paget disease [112,113] or primary hyperparathyroidism [114,115]. Interestingly, ^99m^Tc-radiolabeled butedronate (2,3-dicarboxypropane-1,1-diphosphonate, DPD, Figure 5) [116] is another SPECT imaging agent with the same indications as [^99m^Tc]Tc-MDP and [^99m^Tc]Tc-HMDP but also has a particular role in the detection of cardiac amyloidosis [117,118,119]. Concerning therapy, a bisphosphonate-related derivative with an ethylenediamine tetraphosphonate structure (EDTMP, also named lexidronam) radiolabeled with ^153^Sm (t_1/2_ = 1.9 days; E_β-max_ = 0.81 MeV) has also been used since the late 1980s [120,121] for the relief of pain resulting from bone metastases; two clinical trials demonstrated its efficacy in this indication versus the placebo and its improved toxicity profile compared to ^89^Sr and ^32^P [122,123]. Notably, etidronate was also selected for radionuclide therapy applications after radiolabeling with beta minus-emitting rhenium isotopes, either ^186^Re or ^188^Re [9,10,11,12,13,14,15,16,17,18,19,20,21], but with rather limited clinical use.

### 2.3. From Standard Bisphosphonates to Bifunctional Derivatives Optimized for Nuclear Medicine

**Figure 6:**
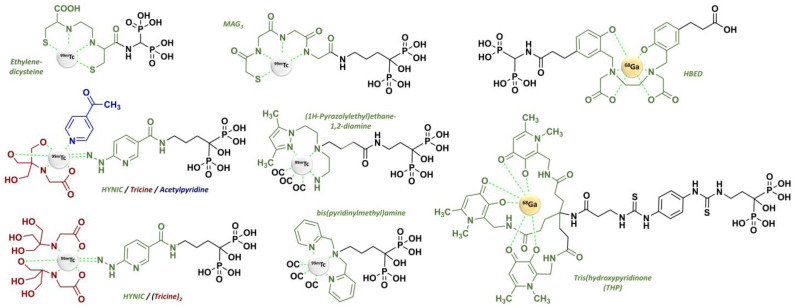
Chemical structure of several bifunctional bisphosphonates conjugated to an acyclic chelator moiety for ^99m^Tc (**left**) or ^68^Ga (**right**) radiolabeling. The oxidation states of ^99m^Tc in these complexes are not shown. The name of each chelating motif is indicated in color.

Beyond the simple, monofunctional bisphosphonate vector molecules mentioned above, other radiopharmaceutical candidates have been designed on the concept of bifunctional agents, dissociating the radiophore part of the molecule that binds to the radioactive atom from the vector part responsible for tropism specificities [106] (Figure 6). In particular, acyclic complexing moieties were used to enable undemanding radiolabeling conditions. Thus, numerous agents for ^99m^Tc radiolabeling were designed, with a chemical variety based on the selected chelating agent. Notably, aminoalkyl derivatives such as pamidronate and alendronate are particularly well-suited to conjugation with a bifunctional chelator because the alkyl chain serves as a spacer and the primary amine serves as an anchoring site for the chelating group. Bisphosphonates bearing an ethylenedicysteine [124], a mercaptoacetyltriglycine (MAG_3_) [125,126], a hydrazinonicotinamide (HYNIC) [125,127], an imidazole- [128], a pyrazole- [129,130] or a pyridine-containing chelator [131] were reported in the literature but were not studied in humans. More recently, a pamidronate derivative with a tris(3,4-hydroxypyridinone) (THP) ligand was reported to allow quantitative ^68^Ga radiolabeling at room temperature and to promise in vivo properties (high bone/muscle and bone/blood ratios and fast blood clearance) comparable to [^18^F]NaF [132]. Lastly, a non-hydroxylated bisphosphonate derivative called P15-041, conjugated to the linear chelator HBED-CC, displayed excellent ^68^Ga labeling properties (>95% radiochemical yield (RCY) at room temperature in 5 min, with a single-vial cold kit formulation developed later [133]), good stability and high bone uptake in normal mice [134]. It was evaluated on humans in early-phase clinical trials for imaging bone metastases in prostate cancer, showing high accumulation in bone lesions and rapid clearance from the blood, soft tissues and normal bone, with tumor-to-normal bone ratios up to 8 at 90 min and reasonable dosimetry estimates [135,136]. A larger clinical study on 51 cancer patients with bone metastases from various types of primary cancers showed the superiority of [^68^Ga]Ga-P15-041 over [^99m^Tc]Tc-MDP in terms of sensitivity, positive predictive value, negative predictive value and accuracy, making P15-041 a valuable PET radiotracer candidate for a wider use in the detection of skeletal metastases [137]. Nevertheless, a direct comparison with another bone-targeting PET radiotracer would be necessary to confirm its efficiency.

**Figure 7:**
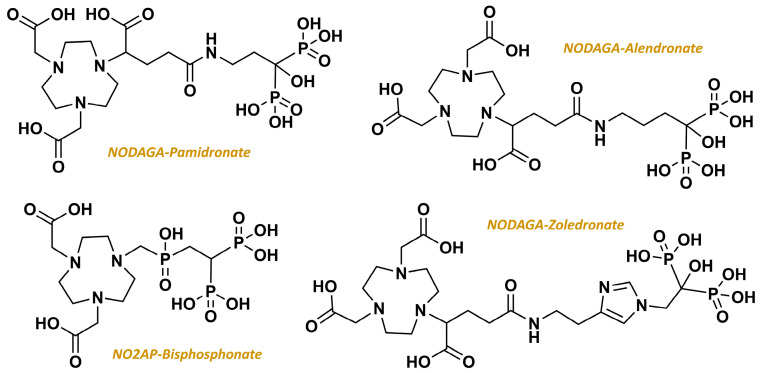
Chemical structure of bifunctional bisphosphonates bearing a nine-membered cyclic chelator, which is particularly suitable for ^68^Ga complexation.

To access other radiometals and to form complexes with high thermodynamic stability, the functionalization of the bisphosphonate derivatives with cyclic chelators was also studied. More specifically, compounds containing a nine-membered cyclic chelator related to NOTA or NODA were designed for easy radiolabeling with ^68^Ga and were used as PET imaging agents (Figure 7). These include NODAGA-Alendronate [138], NODAGA-Pamidronate [139] and NO2AP-Bisphosphonate [140], which has demonstrated high diagnostic efficiency in humans, with bone metastasis detection properties comparable to [^18^F]NaF [141]. Another compound, NODAGA-Zoledronate, also achieved quantitative yields when radiolabeled with ^68^Ga [142] and demonstrated its clinical value in the detection of bone metastases in prostate cancer patients who experienced PSA progression on PSMA-based radioligand therapy or in the setting of restaging [143,144]. NODAGA-Zoledronate also displayed an important uptake in atherosclerotic plaques, which could make it suitable for risk assessment by PET/CT imaging in patients with atherosclerotic cardiovascular disease [145,146]. Lastly, from a radiochemical point of view, it is interesting to note that derivatives with a nine-membered cyclic chelator can be labeled with ^18^F via a non-covalent radiofluorination approach that relies on the formation of [^18^F]aluminum fluoride [147], as exemplified with [^18^F]AlF-NOTA-Pamidronate [148]. Aside from NOTA and NODA derivatives, mesocyclic chelators are a recent group of complexing moieties suitable for ^68^Ga labeling [149]. Derived from 6-amino-1,4-diazepine-triacetic acid, these AAZTA-derived chelators allow fast ^68^Ga radiolabeling under mild conditions. Such a chelator was used to functionalize several bisphosphonate derivatives which could offer promising diagnostic potential, although they have only been evaluated in mice up until now [150].

**Figure 8:**
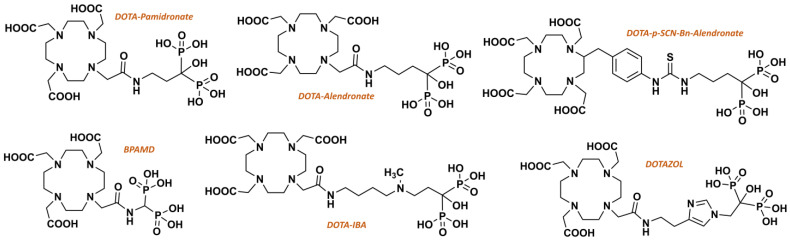
Chemical structure of bifunctional DOTA-bisphosphonates for Me(III) (e.g., ^68^Ga or ^177^Lu) radiolabeling.

The functionalization of bisphosphonates with a DOTA chelator has also been investigated, with a view to developing theranostic approaches based on a unique vector molecule for both diagnostic purposes and therapy (Figure 8). DOTA is a 12-membered macrocyclic chelator characterized by its ability to form thermodynamically and kinetically stable complexes with a variety of radiometals including ^111^In and ^68^Ga for imaging purposes, or ^90^Y and ^177^Lu for TRT applications [32]. One of the simplest DOTA-containing bisphosphonates is the bisphosphonate monoamide analogue of DOTA, BPAMD, which can be prepared via a rather convenient synthesis route [151] and radiolabeled with ^68^Ga in fairly straightforward conditions. It initially demonstrated promising potential for high bone accumulation in mice PET imaging studies [152]. Subsequently, it showed positive results in its first-in-human application, with significantly high target-to-soft tissue ratios and rapid renal clearance [153]. Its standardized uptake values (SUVs) were comparable to [^18^F]NaF, and some metastases even displayed higher bisphosphonate accumulations. These encouraging diagnostic outcomes paved the way for the first therapeutic applications of BPAMD, replacing ^68^Ga with β-emitter ^177^Lu. In this way, the in-house preparation protocols of [^177^Lu]Lu-BPAMD were developed for routine production to treat patients with skeletal metastases [154,155]. After conclusive preclinical evaluation against other simple DOTA-bisphosphonate derivatives (including original dimeric molecules bearing bisphosphonate groups on two of the four acetate arms of the DOTA core) [156], [^177^Lu]Lu-BPAMD was successfully administered to a number of patients, showing a biodistribution similar to its ^68^Ga-labeled counterpart [157,158]. The prolonged contact time of [^177^Lu]Lu-BPAMD with bone metastases resulted in high tumor doses, leading to a significant reduction in osteoblastic activity in bone lesions. Moreover, the therapy did not cause any significant adverse events. In comparison with [^177^Lu]Lu-EDTMP, [^177^Lu]Lu-BPAMD demonstrated higher bone uptake and a higher target-to-background ratio; however, this could be due to the much higher amount of ligand used in the synthesis of [^177^Lu]Lu-EDTMP and its potential target blocking [159]. Although BPAMD displayed good results in both diagnostic and therapeutic applications in humans, the further chemical optimization (potentially requiring a more demanding synthesis route) of such a bisphosphonate-based probe could still be considered. For example, a BPAMD analog bearing a reversible albumin-binding domain was designed to delay body elimination and enhance target accumulation for TRT purposes [160]. More practically, a hydroxy derivative could be used to improve its affinity for the bone matrix, or a nitrogen-containing substituent might be introduced as a spacer, because close proximity of the chelating group to the vector moiety could impair target recognition [161]. The very recent DOTA-ibandronate (DOTA-IBA) was designed on these criteria and displayed both good in vitro characteristics (stability > 91% over 4h after ^68^Ga radiolabeling, high hydrophilicity and 80% plasma protein binding) and in vivo properties (no tissue or organ toxicity in mice, rapid blood clearance with high bone uptake and accurate bone PET/CT images in mice and rabbits); a first-in-patient study of [^68^Ga]Ga-DOTA-IBA also showed comparable results to [^99m^Tc]Tc-MDP in the identification of skeletal lesions, with a higher sensitivity for showing small lesions and a higher target-to-non-target ratio for [^68^Ga]Ga-DOTA-IBA compared to [^99m^Tc]Tc-MDP [162]. In addition, a dosimetry study confirmed its high activity uptake and long retention in bone, with absorbed doses to the whole body and critical organs (red marrow and drug-excretion organs such as kidneys and bladder) within the safety limit [163]. The therapeutic pendant [^177^Lu]Lu-DOTA-IBA was logically developed and could be efficiently radiolabeled after 15 min at 95 °C in a 0.25 M sodium acetate buffer pH 10.4 [164]. The early clinical evaluation of [^177^Lu]Lu-DOTA-IBA (370–1110 MBq) in five patients with bone metastases of malignant diseases showed fast and long-lasting bone pain palliation in three patients, with no detectable hematological or organ toxicity. Subsequently, a phase 0/1 trial on 18 patients with bone metastases first provided dosimetry data consistent with those of [^68^Ga]Ga-DOTA-IBA [165]. Next, the single administration of 891.5 ± 301.3 MBq of [^177^Lu]Lu-DOTA-IBA caused bone pain palliation in 82% of patients and was associated with partial responses in three patients, disease progression in one patient, and stable disease in 14 patients according to the [^68^Ga]Ga-DOTA-IBA PET/CT follow-up at 8 weeks. The highly promising effects of this DOTA-bisphosphonate, radiolabeled with either ^68^Ga for PET imaging or ^177^Lu for TRT, should be confirmed in a larger population but already suggest the theranostic potential of this molecule. Several case reports support these data [166], some even reporting the efficacy of DOTA-IBA radiolabeled with alpha emitter actinium-225 [167].

Overall, although the newly developed candidate DOTA-IBA offers undeniable advantages, its use in humans is still scarcely reported. To date, other molecules have been developed on the same model such as DOTA-Pamidronate [168] and DOTA-Alendronate [169,170,171,172]; however, the optimized theranostic DOTA-bisphosphonate that is most widely used in humans remains DOTAZOL.

## 3. DOTAZOL: Chemistry and Radiochemistry Considerations

DOTAZOL is based on the structure of third-generation hydroxylated amino-bisphosphonate zoledronate, functionalized at position four of the imidazole ring by an ethyl-monoamide-DOTA moiety [173].

### 3.1. Chemical Synthesis of DOTAZOL

**Scheme 1:**
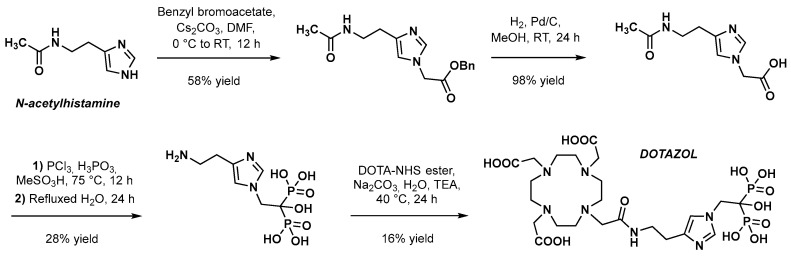
Original synthesis sequence of DOTAZOL as described by Meckel et al. [168].

The first synthesis of DOTAZOL was reported by Meckel et al. in 2017, who presented this macrocyclic bisphosphonate as a promising potential theranostic tool in the management of skeletal metastases [168]. The first step of the synthesis sequence (Scheme 1) consisted of the functionalization of ω-N-acetylhistamine with an acetic arm on the imidazole nitrogen. The use of benzyl bromoacetate with cesium carbonate in DMF formed the desired compound in the average yield via a N-alkylation reaction, followed by a deprotection step catalyzed by palladium on carbon in methanol under hydrogen atmosphere. The hydroxybisphosphonate core was then obtained in a modest yield by reacting a mixture of phosphorus trichloride and phosphorus acid (probably to increase reaction yield compared to PCl_3_ alone [174]) in methanesulfonic acid with the latter 1H-imidazole-1-acetic acid derivative. Interestingly, cleavage of the acetamide to form the primary amine was achieved during the same step, based on the strong acidic condition after the hydrolysis of phosphorus trichloride. Finally, functionalization with the chelating moiety was achieved through the nucleophilic substitution of activated DOTA-NHS-ester in water in the presence of sodium carbonate [169].

**Scheme 2:**
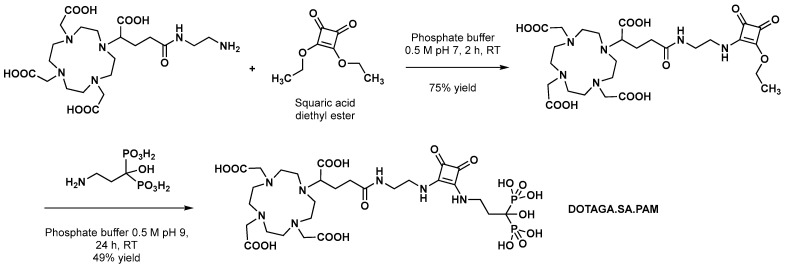
Synthesis sequence of DOTAGA.SA.PAM, a DOTAZOL-related bisphosphonate, as described by Greifenstein et al. [176].

The overall yield of this synthesis sequence is 2.5%, the most limiting steps being the formation of the bisphosphonate motif (28%) and, most importantly, the anchoring of the DOTA group (16%). Regarding this final step, because unprotected bisphosphonates have poor solubility in organic solvents and dissolve only in aqueous buffers, an amide bond formation is not an appropriate strategy because most active esters tend to hydrolyze in aqueous buffers. Thus, the solution proposed here is to use DOTA-NHS-ester; however, it was reported that this compound led to an increased amount of free DOTA as a hydrolysis product of the NHS ester, resulting in successive, laborious final purification cycles. In 2022, Greifenstein et al. proposed a practical and efficient alternative coupling method between the 12-membered macrocycle and the bisphosphonate moiety. This ligation method, which relies on the pH-sensitive reactivity of diethyl squarate, is progressively gaining prominence in the development of novel radiopharmaceuticals [175]. Although the compound that was ultimately obtained was not DOTAZOL (the squaric acid replacing the imidazole ring), the stability of squaric acid esters in aqueous buffers would provide a much more straightforward coupling reaction method (Scheme 2), with no significant formation of side products.

### 3.2. DOTAZOL Radiolabeling with ^68^Ga or ^177^Lu

The initial ^68^Ga radiolabeling of DOTAZOL was performed manually, involving sodium acetate buffer 0.5 M pH 4 and post-processed ^68^Ga in acetone/HCl and 25 nmol ligand; the mixture was heated at 98 °C for 15 min. After terminal purification on a weak anion exchange cartridge, the radiochemical purity (RCP) of [^68^Ga]Ga-DOTAZOL was >98% with the RCY between 80 and 95% [168]. The same method was reported for the preparation of ^68^Ga-labeled DOTAZOL for clinical use [177]. Meisenheimer et al. proposed an alternate ^68^Ga radiolabeling protocol using ammonium acetate 0.08 M with ethanol as a cosolvent, concentrated ^68^Ga in NaCl 5 M pH 1, and heated at 95 °C for 10 min [178]. A thorough study of the reaction conditions was carried out simultaneously and showed that, interestingly, the nature of the reaction vial had a significant influence on the overall reaction outcome. Several polyvalent cations present in the glass could interact with the bisphosphonate molecules, leading to their precipitation and/or adsorption on the reaction vessel. This issue has apparently not been reported in the literature for other DOTA-bisphosphonates; however, it should be considered in the context of unsatisfactory radiolabeling results involving the ionic molecules likely to form this type of interaction [179]. The terminal purification step via solid phase extraction (SPE) was also identified as critical in the [^68^Ga]Ga-DOTAZOL preparation process. The use of a weak anion exchange cartridge to trap the product and then elute it with phosphate buffer saline could be considered [168]; however, the screening of dozens of SPE cartridges did not identify any fully suitable model as thecartridges showed either no retention, the retention of all chemical species, or e thpartial/imperfect retention of one or other species [178]. This parameter therefore requires in-depth study and adaptation to each DOTA-Bisphosphonate used. Indeed, similar issues have been reported with other compounds such as BPAMD, although apparently reliable solutions have been found for this derivative [152,180]. Finally, the automation of the ^68^Ga radiolabeling of DOTAZOL was reported to be quite challenging (notably due to the influence of the reaction vial and to the SPE purification step) [178], which could complicate its use in humans on a larger scale. Thus, the optimized conditions for the automated preparation of [^68^Ga]Ga-DOTAZOL have yet to be identified and adapted to the various GMP-compliant synthesis modules available at present [181]. The ideal scenario for successful and lasting integration into routine clinical practice would probably be the industrial development of DOTAZOL, formulated in a single-vial cold kit for ^68^Ga radiolabeling, which would guarantee the easy preparation of the radiopharmaceutical.

The original ^177^Lu radiolabeling protocol was in line with the usual conditions for this type of reaction [27], involving sodium acetate buffer 0.25 M, [^177^Lu]LuCl_3_ (1 GBq) in 0.04 M HCl and only 20 nmol (~14 µg) DOTAZOL, which were heated at 98 °C for 30 min [168]. In comparison, the ^177^Lu radiolabeling of DOTA-IBA employed the exact same reaction conditions, with a heating time of 15 min. The quantitative complexation yields were reached with this method, exempting it from a final purification step. Khawar et al. later described a modified radiolabeling protocol replacing sodium acetate with ascorbate buffer 1.3 M plus gentisic acid 0.3 M as an anti-radiolysis compound [182]. Although the ^177^Lu activity used was higher (~6 GBq was added to the reaction medium and heated at 95 °C for 30 min), excellent RCP and RCY values were reached (≥98% and ≥95%, respectively); the radiopharmaceutical thus prepared was subsequently administered to humans. This protocol was also applied by both Yadav et al. [183] and Kreppel et al. [184], who gave details of the quantities of the vector used for ^177^Lu radiolabeling (60 µg), which was logically higher than that used for ^68^Ga radiolabeling. Regardless of the radiolabeling conditions, the pH of the reaction medium should be carefully controlled to be around 5, which is ideal for lutetium-177 complexation [27]. It is worth mentioning that these preparations were carried out manually, again raising the issue of the need for pharmacotechnical works aimed at developing efficient and reliable automated synthesis routes in accordance with GMP guidelines. Additionally, although all reported clinical uses of [^177^Lu]Lu-DOTAZOL mention in-house preparation, the industrialized production of this radiopharmaceutical would be a further step toward wider clinical applications.

### 3.3. Quality Controls of Radiolabeled DOTAZOL: A Critical Step

As well as several parameters involved in the radiolabeling of DOTAZOL, the quality control methods for determining th RCP of this ^68^Ga/^177^Lu-labeled bisphosphonate proved to be critical points in the preparation process of such investigational radiopharmaceuticals.

The initial radio-TLC conditions for controlling the RCP of [^68^Ga]Ga-DOTAZOL used an acetone/acetylacetone/HCl mixture (10/10/1) as the mobile phase and silica 60 F254 TLC plates as the stationary phase [168]. With this eluent composition, the unbound ^68^Ga is chelated by acetylacetone to form gallium(III) tris(acetylacetonate), migrating to the solvent front. Colloidal gallium-68 should also convert to ionic [^68^Ga]Ga^3+^ due to its extremely low pH, facilitating its subsequent chelation by acetylacetone and its migration to the solvent front. Notably, slightly adapted analytical conditions (e.g., using acetonitrile instead of acetone) have been reported for other ^68^Ga-labeled bifunctional bisphosphonates [146]. To guarantee the reliability of the results obtained by this analysis and to verify that part of the free gallium identified in radio-TLC does not come from the dissociation of the DOTA-complex [185,186], a threeplate TLC system was later proposed in order to more formally identify all the chemical species potentially present [178]. Table 1 summarizes the migration profiles expected under the different analytical conditions. Interestingly, these conditions were also used to control the RCP of the [^68^Ga]Ga-DOTAZOL used in humans, but only partially, with the single acetylacetone/acetone (1:1) system [177].

Radio-TLC analytical conditions for [^177^Lu]Lu-DOTAZOL tended to be much more straightforward and relied on iTLC plates with the citrate buffer 0.1 M pH 4 [168,183], although a three-plate protocol similar to the one for [^68^Ga]Ga-DOTAZOL was proposed [182]. Overall, in view of the difficulty of discriminating by TLC between two highly hydrophilic chemical entities such as free ^68^Ga^3+^/^177^Lu^3+^ and radiolabeled bisphosphonates, particular attention should be paid to the rigorous validation of the RCP control conditions for each of these different radiopharmaceuticals. In addition, special consideration should be given to the redaction of the experimental protocols provided in scientific publications to ensure maximum reproducibility of such analytical processes.

The HPLC methods for the RCP determination of radiolabeled DOTA-bisphosphonates are considered difficult to implement [176]. For some derivatives, the use of an anion-exchange stationary phase is preferred in order to properly discriminate free radiometal from radiolabeled bisphosphonate [152,180]. Nevertheless, in order to take advantage of the reverse-phase radio-HPLC configuration usually employed for radiopharmaceutical quality controls, several protocols relying on reverse-phase columns were proposed. The original HPLC analysis method of [^68^Ga]Ga-DOTAZOL used a water/acetonitrile gradient and required sample incubation in 0.25 M desferoxamine for 1 min to complex free ^68^Ga^3+^ and discriminate it from the radiolabeled bisphosphonate [168]. However, these conditions made [^68^Ga]Ga-DOTAZOL pass through the column without retention and were therefore unsuitable for the formal identification of the radiolabeling product. An alternative method using an isocratic mixture of tetrabutylammonium phosphate 59 mM and methanol (9:1) as the mobile phase allowed the slight retention of the [^68^Ga]Ga-DOTAZOL complex on the reverse stationary phase [178]. This method was also used for quality control of the ^68^Ga-labeled DOTAZOL evaluated in humans [182]. Finally, an HPLC analysis method for [^177^Lu]Lu-DOTAZOL that is transposable to other phosphonate-containing radiopharmaceuticals was developed by Eryilmaz et al., based on a disodium hydrogen phosphate mobile phase adjusted at pH 1.5–1.8 with orthophosphoric acid and containing N,N-dimethyltetradecylamine [187]. This latter reagent can form ion pairs with phosphonate groups and increase the retention of bisphosphonates via the lipophilic moiety of the alkylamine. In summary, similar to radio-TLC methods, particular attention should be given to the validation of a reliable and efficient radio-HPLC method for the RCP determination of radiolabeled bisphosphonates to guarantee the accurate analysis of the radiopharmaceutical preparation and to avoid either false positive or false negative results.

## 4. Preclinical Investigations on DOTAZOL

### 4.1. [^68^Ga]Ga-DOTAZOL

Only limited data on the non-clinical evaluation of DOTAZOL are available in the literature, reflecting its rapid transfer to clinical use. Initial works on DOTAZOL relate its extremely high in vitro adsorption on hydroxyapatite when radiolabeled with ^68^Ga, which is slightly better than [^68^Ga]Ga-DOTA-Pamidronate and its dehydroxylated analog [^68^Ga]Ga-BPAPD (92.7 ± 1.3% vs. 91.2 ± 2.7% and 83.0 ± 0.8%, respectively) [168]. An ex vivo organ biodistribution study on healthy rats showed high accumulation in bone and low uptake in soft tissues, with 40 to 50% of the injected doses detected in the skeletons at 60 min post-injection (p.i.). Notably, [^68^Ga]DOTAZOL displayed the highest bone-to-blood ratio in comparison with [^68^Ga]Ga-DOTA-Pamidronate and [^68^Ga]Ga-BPAPD (11.5 vs. 7.6 and 3.7, respectively). The in vivo biodistribution experiments were in line with the above results, showing the rapid bone accumulation of [^68^Ga]Ga-DOTAZOL with a plateau level at 45 min p.i. and a fast blood clearance. Interestingly, t hepreclinical evaluation of [^68^Ga]Ga-NODAGAZOL highlighted, in comparison with [^68^Ga]Ga-DOTAZOL, its higher femur accumulation (SUV = 4.53 ± 0.28 vs. 3.67 ± 0.37 at 60 min p.i., respectively). [^68^Ga]Ga-NODAGAZOL also displayed higher tumor-to-background ratios than [^68^Ga]Ga-DOTAZOL (femur-to-blood = 57.5 ± 5 vs. 8.4 ± 1.4 at 60 min p.i. respectively), making it even more suitable than DOTAZOL for diagnostic use. However, this molecule cannot be used for therapy applications, as the NODAGA chelator is not suitable for large radiometals such as ^177^Lu [142].

### 4.2. [^177^Lu]Lu-DOTAZOL

Similar to its ^68^Ga-labeled counterpart, [^177^Lu]Lu-DOTAZOL displayed fast and high uptake in bones and low accumulation in soft tissues, according to the ex vivo biodistribution studies in male Wistar rats [168]. Importantly, the ^177^Lu complex showed longer renal clearance and higher accumulation in the kidneys than [^68^Ga]Ga-DOTAZOL (1.70 ± 0.13% ID/g vs. 0.53 ± 0.04% ID/g at 60 min p.i., respectively), suggesting the importance of close monitoring of the renal function before and during treatment (particularly in the case of high and/or repeated doses). The in vivo evaluation of [^177^Lu]Lu-DOTAZOL was consistent with ex vivo experiments, positioning this investigational radiopharmaceutical as a good candidate for TRT approach in view of its high skeletal accumulation and good target-to-soft tissues ratio.

In the perspective of a potential ^68^Ga/^177^Lu/^225^Ac theranostic triplet, the early preclinical evaluation of DOTAZOL labeled with actinium-225 evidenced high bone uptake in healthy Wistar rats, which was comparable to the ^68^Ga and ^177^Lu-containing derivatives [188]. No clinical signs of toxicity were evidenced over a 2-month observation period; however, the microscopic histopathological analysis of the kidneys 3 months after a single dose of ~400 kBq [^225^Ac]Ac-DOTAZOL revealed significant tubular damage in most of the seven animals studied. As suspected with the ^177^Lu-radiolabeled derivative, DOTAZOL complexed with radiometals for TRT purposes may present a renal toxicity that should be considered and may require concomitant strategies to reduce these adverse effects [189].

## 5. Clinical Uses of DOTAZOL

### 5.1. PET Imaging with [^68^Ga]Ga-DOTAZOL

An early experience with [^68^Ga]Ga-DOTAZOL imaging in humans was reported by Zhang et al. [190], particularly in prostate cancer patients for whom DOTAZOL showed an approximately threefold higher uptake in skeletal lesions than [^68^Ga]Ga-PSMA-11. The introduction of this second-generation bifunctional bisphosphonate follows from the initial use of [^68^Ga]Ga-BPAMD in the same indications and was associated with a perfectible radiosynthesis process, suboptimal accumulation in bone metastases (possibly due to its non-hydroxylated chemical structure) and significant uptake in healthy tissues [191].

**Figure 9:**
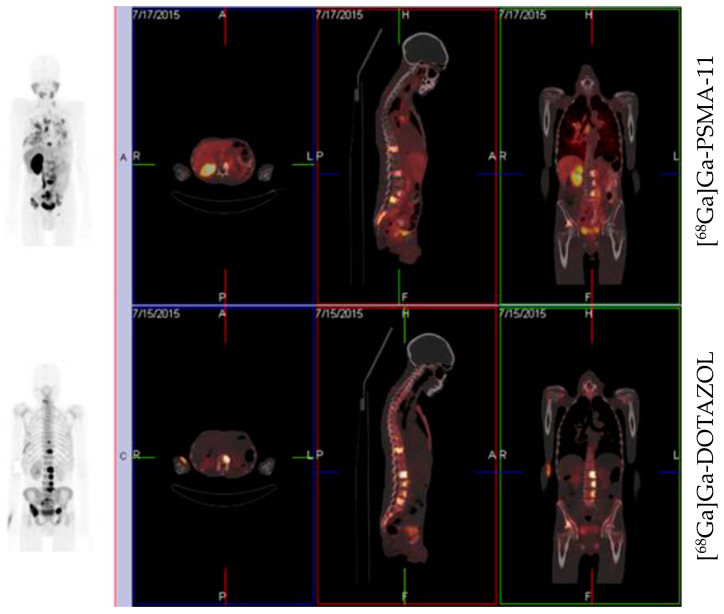
Whole-body PET/CT images acquired 60 min p.i. 170 MBq [^68^Ga]Ga-PSMA-11 and 155 MBq [^68^Ga]Ga-DOTAZOL, respectively, in a single prostate cancer patient (71-years-old, Gleason 4 + 4), as reported by Pfannkuchen et al. [158].

The initial observation of [^68^Ga]Ga-DOTAZOL biodistribution in a single patient with prostate cancer evidenced, in comparison with [^68^Ga]Ga-PSMA-11, a more intense uptake in the bone metastases associated with lower activity in background and in non-target organs (Figure 9) [158]. In this context, in order to better characterize the fate of [^68^Ga]Ga-DOTAZOL in the human body, a larger biodistribution study was carried out on five patients with metastatic breast, bronchial or prostate carcinoma [177]. After the injection of 2.15 ± 0.17 MBq/kg of [^68^Ga]Ga-DOTAZOL, the initial rapid uptake of the tracer in bone could be observed until 30 min p.i., followed by a further gradual rise. The comparison of PET/CT images in the same bronchial carcinoma patient showed a 2.56-fold higher uptake for [^68^Ga]Ga-DOTAZOL than [^18^F]FDG. In addition, a greater number of apparent lesions was found in each patient with the ^68^Ga-labeled bisphosphonate than with the [^18^F]FDG in bronchial carcinoma, the [^68^Ga]Ga-PSMA-617 in prostate cancer and th [^99m^Tc]Tc-MDP in breast cancer, respectively. Dosimetric analysis showed that [^68^Ga]Ga-DOTAZOL, like other bone-seeking agents, delivered the highest radiation-absorbed dose to the bladder (0.368 mSv/MBq), followed by the osteogenic cells (0.040 mSv/MBq), the kidneys (0.031 mSv/MBq) and red marrow (0.027 mSv/MBq). In a theranostic perspective, these high bladder- and kidney-absorbed doses have to be considered but should be easily reduced by proper hydration and rapid diuresis [189].

Aside from this specific study, the clinical use of [^68^Ga]Ga-DOTAZOL is also reported for the selection and follow-up of patients considered for TRT with [^177^Lu]Lu-DOTAZOL [182,183,184].

### 5.2. Targeted Radionuclide Therapy with [^177^Lu]Lu-DOTAZOL

**Figure 10:**
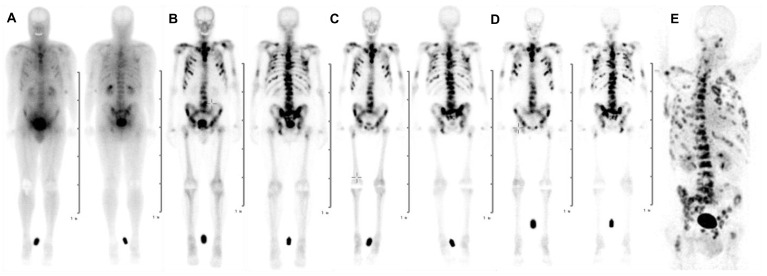
Planar scintigraphy (anterior and posterior views) after the infusion of [^177^Lu]Lu-DOTAZOL at (**A**) 20 min, (**B**) 3 h, (**C**) 24 h and (**D**) 168 h, and (**E**) PET/CT after injection of [^68^Ga]Ga-DOTAZOL in a patient with bone metastases secondary to bronchial carcinoma, as reported by Khawar et al. [182].

The initial assessment of biodistribution and normal organ-absorbed doses after therapeutic doses of [^177^Lu]Lu-DOTAZOL was performed by Khawar et al. in patients with either metastatic prostate cancer (n = 2) or bronchial carcinoma (n = 2) (Figure 10) [182]. After a single mean injected dose of 5968 ± 64 MBq, qualitative SPECT analysis evidenced early and high uptake in the bladder, followed by the kidneys and soft tissues. Consistent with preclinical data, the kidneys showed a rapid decrease in activity at 3 h p.i., concomitantly with an intense and continuous skeletal uptake. Interestingly, despite the small sample size, the skeletal-to-soft tissue contrast values appeared better in bronchial carcinoma patients than in prostate cancer patients. The mean organ-absorbed doses were the highest for the osteogenic cells (3.33 ± 0.35 mSv/MBq), followed by the kidneys (0.49 ± 0.16 mSv/MBq), red marrow (0.46 ± 0.06 mSv/MBq) and the urinary bladder wall (0.32 ± 0.02 mSv/MBq). As a comparison, the dose delivered to the kidneys by [^177^Lu]Lu-DOTAZOL ranged from 1.2 to 1.88 times lower than that of [^177^Lu]Lu-PSMA-617 [192,193].

The early safety data on the clinical use of [^177^Lu]Lu-DOTAZOL were collected by Fernández et al. [194]. After a single mean injected dose of 5780 ± 329 MBq in bone metastatic prostate cancer patients (n = 9), the normalized absorbed doses ranged from 0.206 to 0.564 Gy/GBq for red marrow, from 0.053 to 0.691 Gy/GBq for the kidneys and from 0.635 to 1.980 Gy/GBq for bone surfaces. Assuming a maximum tolerated dose of 2, 23 and 10 Gy for red marrow, kidneys and bone surfaces, red marrow was identified as the dose-limiting organ for all patients, with maximum safely injectable activity ranging from 3.5 to 9.7 GBq (median 6.0 GBq). Regarding the hematological and biochemical parameters, a significant, transitory reduction was observed for leukocytes at 4 and 10 weeks p.i. and for platelets at 4 weeks p.i., with recovery from weeks 4 to 10. Conversely, the treatment had no significant effect on lactate dehydrogenase, alkaline phosphatase, creatinine, hemoglobin or hematocrit levels. No other relevant adverse events such as xerostomia, fatigue, nausea, loss of appetite, nephrotoxicity or hepatotoxicity was experienced by any patient.

The therapeutic safety and efficacy of [^177^Lu]Lu-DOTAZOL was further documented by Yadav et al., who conducted a prospective, single-arm study in 40 patients experiencing bone pain due to skeletal metastases of either prostate (n = 11), breast (n = 23) or lung cancer (n = 6) [183]. Notably, a fixed activity of 1295 MBq (i.e., 2.7 times lower than the most conservative activity of 3.5 GBq recommended by Fernández et al. [194]) was administered as a single-dose treatment in 15 patients, whereas 25 patients received two cycles with a 3-month interval. Overall, 27.5% patients experienced a >70% reduction in the visual analogue scale (VAS) for pain intensity measurement, 50% had a 40–70% decrease in VAS, 12.5% had a 20–40% decrease in VAS and 10% had a <20% decrease in VAS or an increase in pain. In addition, significant improvement was identified in the Karnofski performance scale (pre-therapy = 60 vs. post-therapy = 80) and in the ECOG performance status (pre-therapy = 3 vs. post-therapy = 2). The median time for the initiation of pain relief was ≤7 days and the median duration of sustained response after the last treatment cycle was 3 months. As patients were not treated by any other anticancer treatment during [^177^Lu]Lu-DOTAZOL evaluation (except for eight patients with prostate cancer having hormonal therapy), the median survival from TRT was 13 months (95% CI 10–14 months) and the 1-year survival probability was 55.4%. Regarding toxicity, none of the patients experienced hematologically serious adverse events and only two patients showed grade II transient anemia after [^177^Lu]Lu-DOTAZOL therapy. No adverse renal or biochemical side effects were recorded. In summary, although this cohort study was not randomized and had no comparative arm, it provided more detailed information on [^177^Lu]Lu-DOTAZOL safety and efficacy in the management of metastatic bone pain in several types of cancers. In future similar studies, it would be of interest to investigate other key parameters, such as metastatic burden reduction or the possibility of more retreatment cycles.

Aside from the previously presented studies involving [^177^Lu]Lu-DOTAZOL, a few case reports also exemplify the use of this radioligand therapy in single patients. For instance, a 50-year-old woman with breast cancer previously treated with chemotherapy, radiotherapy and antihormone therapy and displaying extensive bone metastases, was first treated with five cycles of [^177^Lu]Lu-BPAMD (~3.2 GBq/cycle) that led to a partial remission of the disease [190]. Approximately 15 months later, the patient was treated with three cycles of [^177^Lu]Lu-DOTAZOL (~11.5 GBq/cycle with ~5 months between each cycle) that achieved a partial remission of the disease after treatment. Comparably, a 64-year-old man with a poorly differentiated, osseous metastatic adenocarcinoma of the lung who had been previously treated with radiation therapy of bone metastases, systemic chemotherapy and immunotherapy (checkpoint inhibitor nivolumab) received two cycles of ~5.5 GBq [^177^Lu]Lu-DOTAZOL [184]. As expected, the radiopharmaceutical showed intense accumulation in the bone metastases and caused a decrease in osteoblastic activity with no apparent side effects. These two case reports combined with the previously discussed studies suggest that, similarl to the radium-223 that is available for the treatment of prostate cancer bone metastases, [^177^Lu]Lu-DOTAZOL could be an acceptable alternative for the management of skeletal metastases from other types of cancer, e.g., breast or bronchial carcinoma.

## 6. Conclusions

The early diagnosis and management of skeletal metastases can significantly improve patients quality of life as the disease is considered a common cause of morbidity and mortality with a high socio-economic impact. Among the radionuclide therapy alternatives available in nuclear medicine, several options are reserved for the management of prostate cancer bone metastases, as is the case for ^223^Ra and, non-specific for bone metastases, [^177^Lu]Lu-PSMA-617. Combining diagnostic and therapeutic dimensions, the ^99m^Tc-labeled bisphosphonate and the [^153^Sm]Sm-lexidronam pair are currently available. However, the samarium-tetramethylenephosphonate complex presents significant drawbacks, including a moderate clinical impact and the instability of the coordination compound, associated with delivery and storage constraints (freezing between −10 °C and −20 °C). In this context, second-generation bifunctional bisphosphonates such as DOTAZOL offer promising prospects for the diagnosis and treatment—at present, as a last-line, palliative approach—of skeletal lesions in a variety of cancer diseases. Functionalized by a DOTA-chelating group with versatile features, DOTAZOL even offers compatibility with the theranostic triplet ^68^Ga/^177^Lu/^225^Ac, similar to somatostatin analogs [195]. To date, the few results available on the use of DOTAZOL in humans, particularly in relation to its therapeutic aspects, are encouraging regarding both efficacy (especially in the reduction of pain associated with bone metastases) and safety. Other promising DOTA-bisphosphonates such as DOTA-IBA seem to follow a similar trend. Nevertheless, the ^68^Ga/^177^Lu-labeled DOTAZOL theranostic combination will have to find its place among the therapeutic alternatives available to date. In addition, further prospective patient studies would be needed to reliably validate the optimal dosing and frequency of cures and assess the potential of DOTAZOL for the diagnosis and treatment of bone metastases in selected types of cancer.

## Figures and Tables

**Table 1:** Radio-TLC conditions proposed by Meisenheimer et al. for [^68^Ga]Ga-DOTAZOL analysis [178]. The green-colored cells indicate the chemical species identified in each condition, potentially by cross-referencing with other radio-TLC analyses.

Mobile Phase	R_f_ [^68^Ga]Ga-DOTAZOL	R_f_ [^68^Ga]Ga^3+^	R_f_ ^68^Ga-Colloids
TBAP 60 mM/MeOH (9:1)	0.7–0.8	0.1–0.3	0.1–0.2
Citrate buffer pH 4	0–0.1	0.7–1	0.1–0.2
Acetylacetone/acetone (1:1)	0–0.1	0.7–0.8	0–0.1/0.5–0.9

TBAP: tetrabutylammonium phosphate.
